# Association between Plain Water and Sugar-Sweetened Beverages and Total Energy Intake among Mexican School-Age Children

**DOI:** 10.3390/nu8120710

**Published:** 2016-12-18

**Authors:** Teresa Shamah-Levy, Claudia Gabriela García-Chávez, Sonia Rodríguez-Ramírez

**Affiliations:** Centro de investigación en Nutrición y Salud, Instituto Nacional de Salud Pública, Avenida Universidad 655, Cuernavaca, Morelos 62100, Mexico; tshamah@insp.mx (T.S.-L.); gabriela.garcia@insp.mx (C.G.G.-C.)

**Keywords:** plain water intake, energy intake, beverage consumption, children

## Abstract

Water consumption promotes a decrease in total diet energy intake, and one explanation for this fact is the replacement of sugar-sweetened beverages (SSBs) by plain water (PW). The objective of this study was to analyze the association between SSB and PW consumption as a part of the total energy intake. Dietary information was obtained by one 24 h recall of 2536 school-age children who participated in the National Nutrition Survey in Mexico. PW and SSB consumption was measured in mL and servings (240 mL), and consumption was stratified into two levels (<2 and ≥2 servings/day). Linear regression models were used to evaluate the association between PW and SSB consumption in relation to total energy intake. Models were adjusted for age, sex, the proportion of energy obtained from non-beverage food, area of residence, and socioeconomic status (based on information regarding housing conditions and ownership of home appliances). PW consumption at the national level was two servings/day, and was not associated with total energy intake. However, the combination of the high consumption of PW and the low consumption of SSB was associated with less total energy intake (*p* < 0.05). Promoting higher PW and lower SSB consumption provides a useful public health strategy for reducing total energy intake and preventing overconsumption among Mexican school-age children.

## 1. Introduction

Water intake is necessary for life, and it should be the main beverage consumed by the population. Comprising 75% of body weight in infants to 55% in the elderly, it is essential for the maintenance of adequate hydration [1] and cellular homeostasis [2].

In addition to being the major component of the human body, water acts as a medium for numerous metabolic reactions, and assists in transporting nutrients, hormones, waste products and heat throughout the body. Hydration is thus fundamental for maintaining normal physical and cognitive performance [3].

Regarding diet, plain water (PW) consumption promotes a decrease in total energy intake [1,4,5]; it tends to replace caloric beverage intake, and creates a sensation of satiety, assuaging feelings of hunger and the desire to eat [5].

One recommendation for reducing the risk of major chronic disease through dietary changes is to replace sugar-sweetened beverages (SSBs) with PW and unsweetened beverages [6].

Some experimental trials have obtained a reduction in energy intake by replacing SSBs with PW [7]; however, various experimental studies of children have yielded less definitive results. One study showed that drinking PW instead of SSB before meals reduced the total energy intake in the short term, while another study found no such connection [4]. SSB consumption and its relationship to energy intake is well documented [8], but there have been no corresponding studies on the role of PW consumption [2,7].

It has been reported that in young to middle-aged adults without obesity, significant changes in energy intake do not correlate with the presence or absence of PW consumption [7]. On the other hand, some studies have shown that PW consumption is associated with healthier diets and reduced risk of chronic disease [9].

Recent studies have concluded that reducing SSB consumption can prevent weight gain in children [10]. It has been documented that drinking PW instead of SSBs prevents weight gain and obesity in children [4]. However, the consumption of caloric beverages (mainly SSBs) is rising rapidly, particularly in low- and middle-income countries in Latin America [8].

In Mexico, school-age children are one of the population groups with the highest energy intakes from beverages as a proportion of the total energy intake and with a high prevalence of overweight/obesity (34.4%) [11,12].

The Institute of Medicine (IOM) in the USA established the adequate intake (AI) of total water, including all beverages and moisture found in foods (the latter accounts for approximately 20% of intake) [12]. The AI of water for children aged four to eight years was set at 1700 mL/day (which includes approximately 1200 mL for total beverages, leaving 500 mL for moisture). The AI of total water for children aged 9 to 13 years was set at 2100 mL/day (girls) and 2400 mL/day (boys); this includes 1600 mL/day (girls) and 1800 mL/day (boys) of total beverages [13].

In Mexico, school-age children consumed 1254 mL of beverages per capita, in 2006, representing 20.7% of the total energy consumption, including 607 mL of PW [11].

Little evidence exists on PW consumption and energy intake among Mexican school-age children. The aim of this study is therefore to evaluate the association between the consumption of PW and SSBs in relation to total energy intake in Mexican school-age children.

## 2. Materials and Methods

### 2.1. Design and Study Population

Data were obtained from the National Health and Nutrition Survey 2012 (ENSANUT-2012, by its acronym in Spanish), a survey with probabilistic stratified cluster sampling representative at national, regional and urban/rural levels. Data collection was done between October 2011 and May 2012 (details can be consulted elsewhere) [14].

Dietary data were available for urban and rural strata in three regions. The initial sample consisted of 2751 children between five and 11 years having the required dietary information. After running two cleaning efforts to eliminate implausible data, the final study population comprised 2536 children.

Ethical aspects. The survey protocol was approved by the Ethics Commission of the National Institute of Public Health (INSP by its Spanish acronym), with ethic approval code 1108, and informed consent was obtained from the parent or guardian of each participant.

### 2.2. Data Collection and Variable Construction

Dietary assessment. Dietary data were collected using a 24 h recall. Complete information on foods and beverages consumed the day before the interview was obtained following a multi-pass method: (1) compiling a preliminary list of foods consumed throughout the day; (2) reviewing the food list to ensure the inclusion of foods often overlooked; (3) completing details on the list of foods such as meal times and associated activities; (4) completing details on each food item, including portion size and recipes; and (5) performing a final review. The details of this method have been published previously [15].

In children younger than 10 years, reporting was done by the mother, caregiver or person in charge of feeding the child; in children 10 years and older, the report was completed by the child assisted by the person responsible for feeding him or her. This information was then supplemented by the child to account for food consumed outside of the home.

### 2.3. Formation of Beverage Groups

Grouping was based on caloric contribution and sugar content, with PW taken as a group despite its lack of energy contribution. A total of six groups was created: (1) PW: included just the water consumed alone (without another ingredient); (2) dairy beverages without sugar: whole milk from any animal species; (3) dairy beverages with sugar: *atole* and *pozol* (traditional Mexican beverages made from corn), among others; (4) non-dairy beverages with sugar: *aguas frescas* (a mixture of fruit or flower, sugar and water), industrialized juices, coffee and tea with sugar, and soft drinks, among others; (5) natural juices; and (6) non-dairy beverages without sugar, listed in Table 1.

### 2.4. Identification of Outliers

To reduce systematic errors, implausible data were excluded from analysis according to two criteria. The first was excessive consumption (mL): we used data distribution to determine the largest consumption amounts in the six beverage groups, and established these values as cutoffs. For the groups concerning PW as well as non-dairy beverages with sugar and natural juices, excessive consumption was defined as anything above 3000 mL; for dairy beverages without sugar, anything above 1000 mL; for dairy beverages with sugar, anything above 1400 mL; and for non-dairy beverages without sugar, anything above 900 mL. We excluded 22 children in this step of the cleaning process. The second criterion was energy consumption (kcal): we defined cutoffs as the mean energy consumption in each group plus three times its standard deviation. Cutoffs were 301.47 kcal for dairy beverages without sugar; 585.32 kcal for dairy beverages with sugar; 669.12 for non-dairy beverages with sugar; 494 mL for natural juices; and 66.51 kcal for non-dairy beverages without sugar. We excluded 193 children in this final step of the cleaning process.

Energy intake. We estimated consumption and the corresponding energy intake for the food items within each group, and calculated the total energy intake for each group as well as the proportion of intake pertaining to each participant. Energy values were based on the food-composition tables formulated by the National Institute of Public Health (Nutrient Data Base, Compilation of the National Institute of Public Health, unpublished material, 2012).

Socio-demographic variables. The age, sex, area of residence and socioeconomic status (SES) of the sample children were obtained by means of a household questionnaire.

Area of residence was defined as rural for localities with <2500 inhabitants and urban for localities with ≥2500 inhabitants.

An SES index was constructed based on housing conditions (flooring and roofing materials); ownership of home appliances (refrigerator, stove, washing machine, television, radio, video player, telephone, and computer); and the number of rooms in the house. We used the Principal Component Analysis to generate a continuous variable which we then divided into tertiles representing low, middle, and high SES categories.

### 2.5. Statistical Analysis

To describe our analytic sample, we estimated percentages for each demographic variable and divided the results into quartiles (p25, p50 and p75) representing the consumption (mL), energy contribution and energy percentage levels of each beverage group.

In view of the biased distribution of beverage consumption and the small size of our consumer sample, we traced the differences on box plots.

To better interpret the association between beverage consumption and total energy intake, we expressed beverage consumption as servings of 240 mL for both PW and SSBs. SSBs were taken from two groups: dairy and non-dairy beverages with sugar.

In addition, we divided beverage consumption quality into four categories: (1) low water (<two servings) and high SSB consumption (≥two servings); (2) high water (≥two servings) and high SSB consumption (≥two servings); (3) low water (<two servings) and low SSB consumption (<two servings); and (4) high water (≥two servings) and low SSB consumption (<two servings). We used two servings as the cutoff point because this value was the median consumption portion for PW and SSBs.

We used two linear regression models to analyze the association between beverage consumption and total energy intake, adjusting by age, sex, area of residence, SES and energy obtained from non-beverage foods. The first model included PW and SSB consumption as continuous variables. Because the relationship between SSBs and total energy intake is not linear, we included the quadratic term SSBs to the model. The second model included PW and SSB consumption in the four categories mentioned below.

In order to maintain the original representativeness levels, we performed all of the analyses using STATA 14.1 SVY module software for survey data.

## 3. Results

### 3.1. Sample Characteristics

After excluding the outliers in beverage consumption and applying the expansion factor, our final analytic sample reached 2536 school children aged five to eleven years, representing a universe of 18,448,445 individuals nationwide.

Characteristics of the sample subjects are described in Table 2. Their mean age was 8.18 ± 2 years (not shown in the Table). Approximately half were male and half were female. About a third (28%) lived in rural areas. Twenty-nine percent occupied the highest and 35% the middle and low SES tertiles.

### 3.2. Beverage Consumption

Table 3 illustrates the total energy consumption by socio-demographic variable. The national median was 1633 kcal/day. Caloric intake among urban dwellers was 1225 kcal/day for the 25th percentile and 2187 kcal/day for the 75th percentile. Rural dwellers showed intakes of 1182 and 2090 kcal/day, respectively.

The median energy intakes in the three SES categories listed in ascending order were 1561, 1701 and 1648 kcal/day. The highest energy intake occurred in the middle SES category with 2264 kcal/day; the lowest intake fell into the low category with 1189 kcal/day.

Table 4 displays the percentage of consumers at the national level and consumption (mL) in medians as well as the 25th and 75th percentiles of each beverage group. It also shows the energy contribution and intake distributions of the groups among school-age children. More than 70% of the children reported consumption of PW, almost 80% consume non-dairy beverages with sugar and more than one-third consume dairy beverages with sugar. Just 2.2% of the children consumed natural juices. The median consumption for PW was 480 mL, or two servings/day, as opposed to barely over one serving/day for both the dairy beverages without sugar and dairy beverages with sugar groups. Consumption reached approximately two servings/day for the non-dairy beverages with sugar, and less than one serving/day for the non-dairy beverages without sugar group.

Dairy beverages with sugar contributed the highest percentage of energy at the national level with a median of 12.2% of total energy intake. However, SSBs (combining dairy and non-dairy beverages with sugar) contributed approximately 20% (data not shown in table).

School-age children in rural areas consumed the highest percentage of non-dairy beverages with sugar (448 mL/day), while their urban counterparts consumed the highest percentage (21.0%) of SSBs in general.

Similarly, as regards the SES of our sample children, children with middle SES had the highest consumption of non-dairy beverages with sugar (427 mL/day), while the high SES group had the highest consumption of dairy beverages with sugar (272 mL/day), as well as the highest percentages of energy from dairy and non-dairy beverages with sugar (14.7% and 8.7%, respectively).

No difference was observed between areas of residence regarding consumption, energy contribution or percentage of energy.

Table 5 presents the association between beverage consumption and total energy intake. PW consumption was not associated with energy intake, whereas SSB consumption showed a positive association amounting to a 69 kcal (*p* < 0.05) per serving increase. The association proved even higher on analyzing the beverage consumption categories. The association of high water–low SSB consumption was 230 kcal less than that of low water–high SSB consumption with regard to total energy intake (*p* < 0.01).

## 4. Discussion

Based on data from a nationally representative survey, our study sheds light on the relationship of PW and SSB consumption with energy intake among Mexican school-age children. The main finding was that the combination of low PW and high SSB consumption was associated with higher total energy intake. We found no association between PW consumption (independent of SSB consumption) and total energy intake.

The median energy intake (1633 kcal/day) was within the required range for school-age children: 1200–2200 kcal/day, depending on the age and physical activity level [16] of the particular child.

Malik et al. found that, in Mexico, all age groups combined received ≈10% of their total energy intake from SSBs. Since then, the proportion of SSB energy intake has increased considerably among individuals older than five years of age [17].

It is important to note that the higher association between beverage consumption and total energy intake was found when combined specifically with high water and low SSB consumption. It has been argued that replacing SSBs with non-caloric beverages or PW may be a useful strategy for weight reduction as a result of less energy intake [18,19].

Our findings are consistent with those of Martinez et al., whose samples of children and adolescents in Uruguay, Brazil and Mexico demonstrated that the highest contribution to the total fluid intake came from beverages containing sugar (i.e., juices and sweet beverages) [20].

As indicated in other studies, one explanation for the observed link between SSB consumption and higher total energy intake may lie in the fact that individuals do not sufficiently reduce their energy intake from other sources to offset the calories ingested from beverages [21,22].

A meta-analysis on the effects of soft drink consumption obtained similar evidence [23]. It also found that replacing sweetened caloric beverages with PW was associated with a sustained caloric deficit among women in a 12-month clinical weight loss trial [19].

A study of Brazilian students from 10 to 11 years old provided no evidence of PW consumption having a protective effect on Body Mass Index increase—children who reported high PW consumption also reported a high intake of other beverages [24]. However, the study did confirm that consumption of juice drinks was a risk factor for increased BMI [24].

According to another study of Mexican children in the same age group as ours, the trends obtained by several dietary intake surveys point to a sharp increase in caloric beverage consumption among pre-school and school-age children. The surveys also found that beverages such as whole milk and sugar-sweetened juices were important contributors to the increased energy intake among children. Mexican school children were found to consume 20.7% of their energy from caloric beverages. The three most commonly consumed were whole milk, fruit juice with various sugar and water combinations, and carbonated and noncarbonated sugared beverages [11].

Findings from several clinical trials, epidemiological studies and intervention initiatives suggest that PW plays a potentially important role in reducing energy intake and, consequently, in preventing obesity [8]. Similarly, the results of a meta-analysis by Popkin et al. on the effects of PW intake alone suggest that PW consumption is linked to reduced energy intake when replacing sugar-sweetened beverages, juice, milk and diet beverages. These findings come primarily from clinical feeding studies, a well-regarded random controlled school intervention, and several additional epidemiological and intervention studies [2].

Our study did not yield the same results in this area, a difference that may be attributable to differences in the methodology for assessing PW intake, as has been the case in other published studies.

Our study has the following limitations that should be addressed in future epidemiological studies: ENSANUT 2012 did not track physical activity for all age groups. Therefore, it was impossible to adjust the models constructed to assess the association between beverage consumption and total energy intake for physical activity, a variable which could be attenuating the association [25,26].

Variability in the consumption of beverages and water in our study might be related to season. The ENSANUT was conducted from October 2011 to May 2012, with varying weather (temperature) throughout the study period potentially contributing to different liquid intakes. Subsequent studies are therefore necessary to determine beverage intake by season.

Moreover, research on child caloric beverages suggests that caloric beverages consumed by school-age children may be underestimated, particularly those consumed at school (e.g., fruit juices and *aguas frescas*) [10].

Another limitation of our study concerns the fact that data analyzed on the consumption of SSBs and PW were collected only from one 24 h recall. Self-reported intake may have been affected by recall bias and/or reporting errors. Such measurement errors could be randomly distributed across the sample or might affect certain sub-populations systematically [27].

Nevertheless, our study also has its strengths, one of which is the fact that the data come from a survey representative at national, state, and urban/rural area levels. This provides a sample size adequate not only to offer precise outcomes and external validity, but also to allow for extrapolating the results to all Mexican school-age children.

Another strength lies in the fact that dietary information was collected using the multi-pass 24 h recall method, thereby increasing accuracy in estimating the usual intake distributions [28]. Additionally, the questionnaire included PW intake as part of the 24 h recall.

The past 30 years have witnessed a marked increase in SSB consumption around the world. As indicated by our study, SSB consumption may account for the increase in energy intake among school-age children and could also be associated with obesity.

Public health initiatives in various countries, Mexico among them [29], are actively promoting PW consumption to help control weight. A number of American associations and organizations recommend drinking water either in greater volume or in place of other beverages as a part of weight management [30].

Effective strategies are required to restrict the supply of sugar-sweetened beverages and other high-calorie, nutrient-poor food products in places frequented by children such as schools, parks and recreation areas. The government needs to implement measures such as taxation in its battle against consumption of these addictive foods and beverages. At the same time, free PW must be made widely available for public health purposes.

## 5. Conclusions

Slashing SSB consumption can provide an important strategy for eliminating excess caloric intake; however, the choice of a replacement beverage is crucial. Promoting PW consumption could be a useful public health policy in that it is clearly an appropriate SSB replacement choice that is also economical if tap water is used. It could also be an effective strategy for balancing energy/nutrient intake and preventing overconsumption among Mexican school-age children.

## Figures and Tables

**Table 1:** Classification of beverage groups among Mexican school-age children (five to eleven years old), ENSANUT 2012.

	Beverage Group	Beverage
Group 1	Plain water (PW)	Plain water
Group 2	Dairy beverages without sugar	Whole milk or soy milk without sugar
		High-fat dairy drinks
		Whole lactose-free milk
		Reduced-fat milk
		Light milk formula
Group 3	Dairy beverages with sugar	Whole milk with sugar
		*Atole* (base on milk, any flavor)
		Yogurt drinks
		Milk-based smoothies
Group 4	Non-dairy beverages with sugar	*Aguas frescas* (a mixture of fruit or flower, sugar and water)
		Energy and sports drinks
		Chocolate drinks made with sugar and water
		*Atole/pozol* (traditional Mexican beverages made with corn flour, water, and sugar)
		Coffee or tea with sugar
		Industrialized juices
		Soft drinks of any flavor
Group 5	Natural juices	Natural fruit juices
		Natural vegetable juices
Group 6	Non-dairy beverages without sugar	Light soft drinks of any flavor
		Mineral water
		Light flavored water
		Powdered sugar-free drinks
		Coffee or tea without sugar

All groups were established according to caloric contribution and sugar content.

**Table 2:** Socio-demographic characteristics of Mexican school-age children (five to eleven years old). National Survey of Health and Nutrition 2012, Mexico.

	*n*	%	95% CI
Sex			
Male	1289	50.1	47.4–52.9
Female	1247	49.8	47.0–52.5
Age (years)			
5	335	12.2	10.5–13.9
6	354	12.4	10.5–14.4
7	406	14.5	12.7–16.3
8	386	14.2	12.4–15.9
9	378	13.9	12.0–15.9
10	332	15.3	13.1–17.4
11	345	17.2	14.9–19.5
Area ^1^			
Urban	1556	71.4	69.4–73.4
Rural	980	28.5	26.5–30.5
Socioeconomic Status ^2^			
Low	977	35.3	32.4–38.1
Middle	899	35.1	32.3–38.0
High	670	29.5	26.3–32.6
Total simple ^3^	2536		

^1^ Rural: <2500 inhabitants; urban: ≥2500 or more; ^2^ Calculated using principal components analysis; includes household characteristics, goods and services; ^3^ *N* = 18,448,445, which represents 18,448,445 school-age children.

**Table 3:** Total energy intake according to socio-demographic variables *.

	Total Energy Intake (kcal/Day)		
	p25	p50	p75
National	1215.3	1632.8	2173.2
Area			
Urban	1224.9	1655.9	2187.2
Rural	1182.2	1582.5	2089.7
Socioeconomic status			
Low	1189.5	1561.3	2149.1
Middle	1269.4	1701.1	2264.1
High	1218.2	1647.7	2145.3

All values shown in this table apply to the total sample. * *n* = 2536, which represents 18,448,445 school-age children.

**Table 4:** Quartiles of consumption, energy contribution and percentage of energy of beverage groups by socioeconomic status and area of residence in Mexican school-age children (*n* = 2536).

	NATIONAL				AREA						SOCIOECONOMIC STATUS								
					Urban			Rural			Low			Middle			High		
	% *	p25	p50	p75	p25	p50	p75	p25	p50	p75	p25	p50	p75	p25	p50	p75	p25	p50	p75
Consumption (mL)																			
Plain water	74.1	240.0	480.0	720.0	240.0	480.0	720.0	240.0	480.0	720.0	240.0	480.0	720.0	240.0	480.0	720.0	240.0	480.0	600.0
Dairy beverages without sugar	27.0	240.0	246.7	370.4	240.0	246.7	370.0	240.0	246.7	411.2	240.0	246.7	400.9	240.0	246.7	380.0	240.0	246.7	370.0
Dairy beverages with sugar	33.2	223.0	258.0	368.0	225.2	259.0	361.2	212.1	256.5	369.4	220.0	255.7	342.4	196.6	250.0	308.7	230.4	272.0	460.0
Non-dairy beverages with sugar	78.9	240.0	408	663.8	240	402.8	625.2	240.0	448.6	705.6	235.0	379.4	703.1	240.0	426.7	643.0	240.0	416.0	605.0
Natural juices	2.2	193.0	252.0	252.0	210.0	252.0	252.0	131.2	252.0	262.5	126.0	252.0	262.5	252.0	252.0	262.5	210.0	252.0	252.0
Non-dairy beverages without sugar	10.1	188.0	225.6	295.2	156.5	225.6	295.2	196.8	225.6	285.7	164.0	225.6	360	196.8	225.6	240.0	123.0	225.6	295.2
Energy contribution																			
Plain water		-	-	-	-	-	-	*-*	*-*	*-*	*-*	*-*	*-*	-	-	-	-	-	-
Dairy beverages without sugar		118.3	143.3	177.5	118.3	143.3	177.3	135	143.3	179.2	118.6	143.3	179.2	118.3	143.3	179.2	118.3	143.3	170.4
Dairy beverages with sugar		148.8	209.1	311.9	158.7	218.9	311.8	117.4	184.4	311.9	114.0	184.4	267.5	131.2	196	279.4	184.4	251.3	349.2
Non-dairy beverages with sugar		89.0	140.7	230.0	89.7	145.6	227.1	76.8	128.8	241.0	74.8	126.2	240.5	89.7	145.4	229.9	98.6	147.6	224.5
Natural juices		74.8	113.4	113.4	94.5	113.4	113.4	56.7	113.4	118.1	56.7	113.4	118.1	113.4	113.4	118.1	94.5	113.4	113.4
Non-dairy beverages without sugar		0.0	0.0	2.4	0.0	0.0	2.4	0.0	0.0	2.5	0.0	0.0	3.2	0.0	0.0	2.4	0.0	0.0	2.4
Energy percentage ^†^																			
Plain water		-	-	-	-	-	-	*-*	*-*	*-*				-	-	-	-	-	-
Dairy beverages without sugar		5.6	8.7	12.8	5.7	8.7	13.2	6.2	9.1	13	6.7	8.8	13.8	5.7	8.9	13.8	5.1	8.5	12.4
Dairy beverages with sugar		8.0	12.2	19.1	8.4	12.7	19.3	6.3	10.7	17.4	6.6	11.2	16.2	7.1	11.4	16.3	10.0	14.7	22.0
Non-dairy beverages with sugar		5.0	8.4	13.5	5.0	8.3	13.3	5.0	8.3	13.4	4.3	8.2	13	5.2	8.1	13.6	5.4	8.7	12.9
Natural juices		4.7	6.4	8.7	4.7	6.4	8.7	4.5	5. 7	8.7	2.6	6.4	9.2	5.1	6.7	8.7	4.7	5.8	8.6
Non-dairy beverages without sugar		0.0	0.0	0.1	0.0	0.0	0.1	0.0	0.0	0.2	0.0	0.0	0.2	0.0	0.0	0.1	0.0	0.0	0.0

All values pertain only to consumers. * Percentage of consumers at national level. ^†^ Regarding total energy intake.

**Table 5:** Multivariate linear regression analyses showing the association between beverage consumption and total energy intake in Mexican school-age children (*n* = 2536).

	Variable	Coefficient (SE)	*p*
Model 1 ^a^	PW consumption (servings)	−1.09 (2.21)	0.611
	SSB consumption (servings)	112.08 (5.75)	<0.001 *
	SSB consumption (servings) ^2^	−6.66 (0.82)	<0.001 *
	Age (years)	−2.88 (2.00)	0.150
	Sex (boy = 1)	1.45 (7.18)	0.839
	Energy from food (kcal)	0.99 (0.01)	<0.001 *
	SES low ^b^	Reference	
	SES Medium	18.37 (9.01)	0.042 *
	SES High	38.75 (10.49)	<0.001 *
	Area of residence (urban = 1)	30.36 (7.99)	<0.001 *
	Constant	73.96 (21.69)	
Model 2 ^c^	Low water–high SSBs ^d^	Refererence	
	High water–high SSBs	−1.009 (14.64)	0.940
	Low water–low SSBs	−218.72 (11.16)	<0.001 *
	High water–low SSBs	−228.71 (12.48)	<0.001 *
	Age (years)	−1.88 (2.15)	0.379
	Sex (boy = 1)	1.65 (7.86)	0.833
	Energy from food (kcal)	1.00 (0.05)	<0.001 *
	SES low	Reference	
	SES Medium	17.71 (9.07)	0.051
	SES High	36.03 (11.17)	<0.001 *
	Area of residence (urban = 1)	34.41 (8.69)	<0.001 *
	Constant	353.53 (23.75)	

^a^ Model 1. Linear regression model with consumption of PW (plain water) and SSBs (sugar-sweetened beverages) as continuous variables, adjusted by age, sex, energy from non-beverage foods, socioeconomic status and area of residence. The quadratic term SSBs was included; ^b^ Socioeconomic status categories calculated using principal components analysis; includes household characteristics, goods and services; ^c^ Model 2. Linear regression model with consumption of water and SSBs as four categories of consumption, adjusted by age, sex, energy from non-beverage foods, socioeconomic status and area of residence; ^d^ Cutoff point for water and SSB consumption was: (1) low water (<two servings) and high SSB consumption (≥two servings); (2) high water (≥two servings) and high SSB consumption (≥two servings); (3) low water (<two servings) and low SSBs (<two servings) consumption; and (4) high water (≥two servings) and low SSBs (<two servings) consumption. * Significant difference (*p* < 0.05).
